# Low-carbon economy transition, environmental policy stringency, and public health: the roles of technological development and air quality

**DOI:** 10.3389/fpubh.2026.1955444

**Published:** 2026-10-07

**Authors:** Wenjuan Bao, Bo Pang

**Affiliations:** 1 School of Economics and Management, Yantai Institute of Technology, YanTai, China; 2 School of Economics and Management, College of Science and Technology Changchun, Changchun, China

**Keywords:** air quality, EPSI, green innovation, life expectancy, low-carbon transition, public health, sustainable development

## Abstract

The transition to a low-carbon economy is increasingly important for climate mitigation, environmental sustainability, and population health. This work examines the long-run relationships between the low-carbon economy transition, represented through its policy dimension by environmental policy stringency (EPSI) and its energy-transition dimension by renewable energy consumption (REC), industrial-process carbon emissions (CO2IP), research and development expenditure (RDE), air quality, healthcare expenditure (CHE), and life expectancy in 16 developed economies over the period 2000–2020. The long-run relationships are estimated using a fixed effects model with DKSEs. We use a Prais–Winsten regression with PCSEs as a robustness check. The outcomes propose that EPSI and REC are positively and statistically significantly associated with life expectancy in the baseline model. CHE is positively correlated, but the correlation is relatively weak. CO2IP is positive and statistically significant in the baseline model, whereas RDE and PM2.5 are statistically insignificant. The robustness analysis further reinforces the positive associations of EPSI and REC with life expectancy, while CHE remains positively significant and PM₂.₅ becomes negative and statistically significant. In contrast, CO2IP and RDE are statistically insignificant in the PCSE specification. To summarize, EPSI and REC emerge as the most consistently supported dimensions of the low-carbon economy transition associated with higher life expectancy. Evidence for industrial-process carbon emissions and RDE is less robust, while the negative association of PM₂.₅ receives stronger support in the robustness analysis. These findings inform environmental, energy, and public-health policy; however, rather than being causal consequences, they should be understood as connections.

## 1 Introduction

The global pursuit of sustainable development increasingly requires countries to address the interconnected challenges of climate change, fossil-fuel dependence, environmental degradation, and public health (1, 2). Rapid industrialization and economic development have increased energy demand, while continued reliance on conventional fossil fuels has contributed to rising greenhouse gas emissions and environmental pressures (3, 4). The consequent degradation in environmental quality is especially critical from a health standpoint due to the release of harmful air pollutants through emissions associated with energy production and through industrial operations (5, 6). Therefore, the move toward a low-carbon economy has assumed increasing importance not only for reasons of mitigating climate change but also for improving environmental quality and safeguarding human health (7, 8). In this study, the low-carbon economy transition is conceptualized through two complementary dimensions: the policy dimension, represented by environmental policy stringency (EPSI), and the energy-transition dimension, represented by renewable energy consumption (REC).

Among the environmental consequences associated with fossil-fuel use and carbon-intensive economic activities, air pollution represents a particularly important pathway through which environmental degradation affects human health (9, 10). Poor air quality increases population exposure to contaminants such as particulate matter (PM₂.₅), nitrogen oxides (NOₓ), sulphur dioxide (SO₂), and ground-level ozone. Such exposure is associated with respiratory and cardiovascular diseases and premature mortality, making air quality an important link between environmental sustainability and public health (11, 12). Consequently, policies that reduce dependence on carbon-intensive energy sources and promote cleaner production and energy systems may generate health co-benefits alongside reductions in environmental pressures (13). Carbon emissions from industrial processes go on to incorporate the pressure placed on the environment due to the carbon-based industry and thus add another perspective to the association between industrialization and human well-being (14).

These interconnected issues have led governments to adopt tougher environmental policies aimed at decreasing emissions and encouraging the production of cleaner products while accelerating the shift toward low-carbon energy systems (15, 16). Environmental Policy Stringency (EPSI) represents an important policy mechanism through which governments can influence firms’ ecological behavior by strengthening environmental regulations, carbon pricing mechanisms, and emissions standards (17, 18). More stringent environmental policies can create incentives for firms to adopt cleaner technologies, boost energy efficiency, and increase investment in ecologically friendly production processes (19, 20). At the same time, however, stringent regulation may impose compliance costs and potentially constrain economic activity, particularly in pollution-intensive industries (21, 22). Therefore, an important research question is whether stronger environmental policy stringency can simultaneously support the low-carbon transition and generate improvements in public health (23). Renewable energy consumption complements this policy dimension by reflecting the extent to which the energy system shifts toward cleaner and lower-carbon energy sources (24, 25).

Research and development expenditure and improved air quality provide important mechanisms through which environmental policies may generate public-health benefits (26, 27). Ecological regulations can stimulate firms to develop and adopt cleaner technologies, while the resulting improvements in production efficiency and energy use can contribute to lower emissions and better environmental quality (28). R&D expenditure represents broader national research, technological development, and innovation capacity that may support technological upgrading, productivity improvement, and the diffusion of more efficient production processes (29). Improved air quality can reduce population exposure to harmful pollutants and consequently improve health outcomes (30, 31). Despite these potentially interconnected pathways, existing research has often examined environmental policy, low-carbon development, research and technological capacity, industrial carbon pressure, air quality, and health outcomes separately. This creates a need for an integrated empirical assessment of whether environmental policy stringency and the transition toward a low-carbon economy are associated with better public health, and whether R&D expenditure, industrial-process carbon emissions, air quality, renewable energy consumption, and healthcare expenditure help explain these relationships.

Against this background, Figure 1 demonstrates the variation in Environmental Policy Stringency across the selected nations over the study span. The variation in policy stringency highlights differences in the intensity and evolution of environmental regulation across countries and provides an important contextual basis for examining whether stronger environmental policies are associated with improved public-health outcomes.

**Figure 1:**
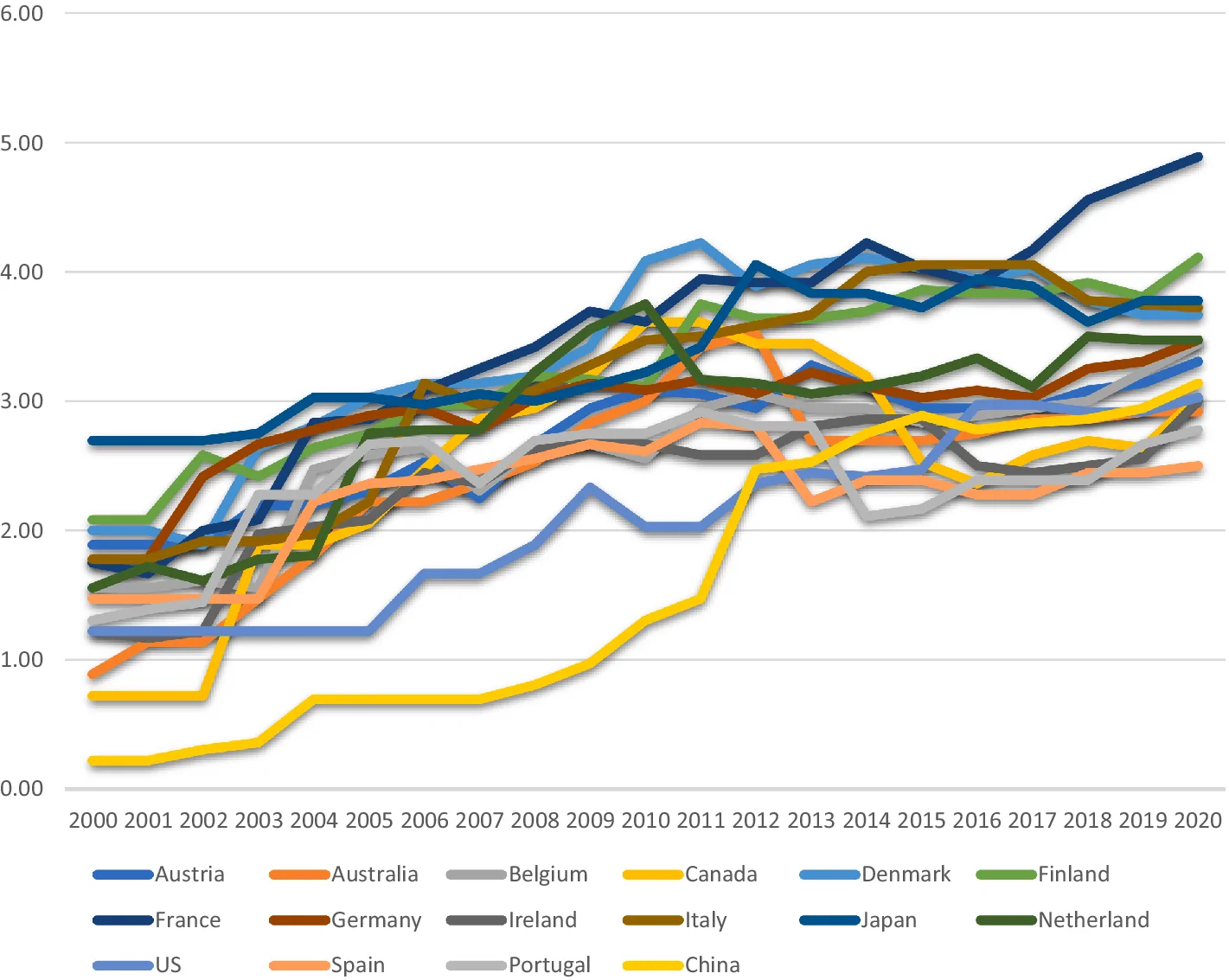
Variability in EPSI across all selected countries from 2000 to 2020 (Source: OECD Environmental Policy Stringency Database; authors’ presentation).

Figure 2 illustrates a steady increase in life expectancy. The consistent upward trend suggests continuous progress in public health, which may be associated with improved healthcare services, cleaner environmental conditions, and sustainable development initiatives.

**Figure 2:**
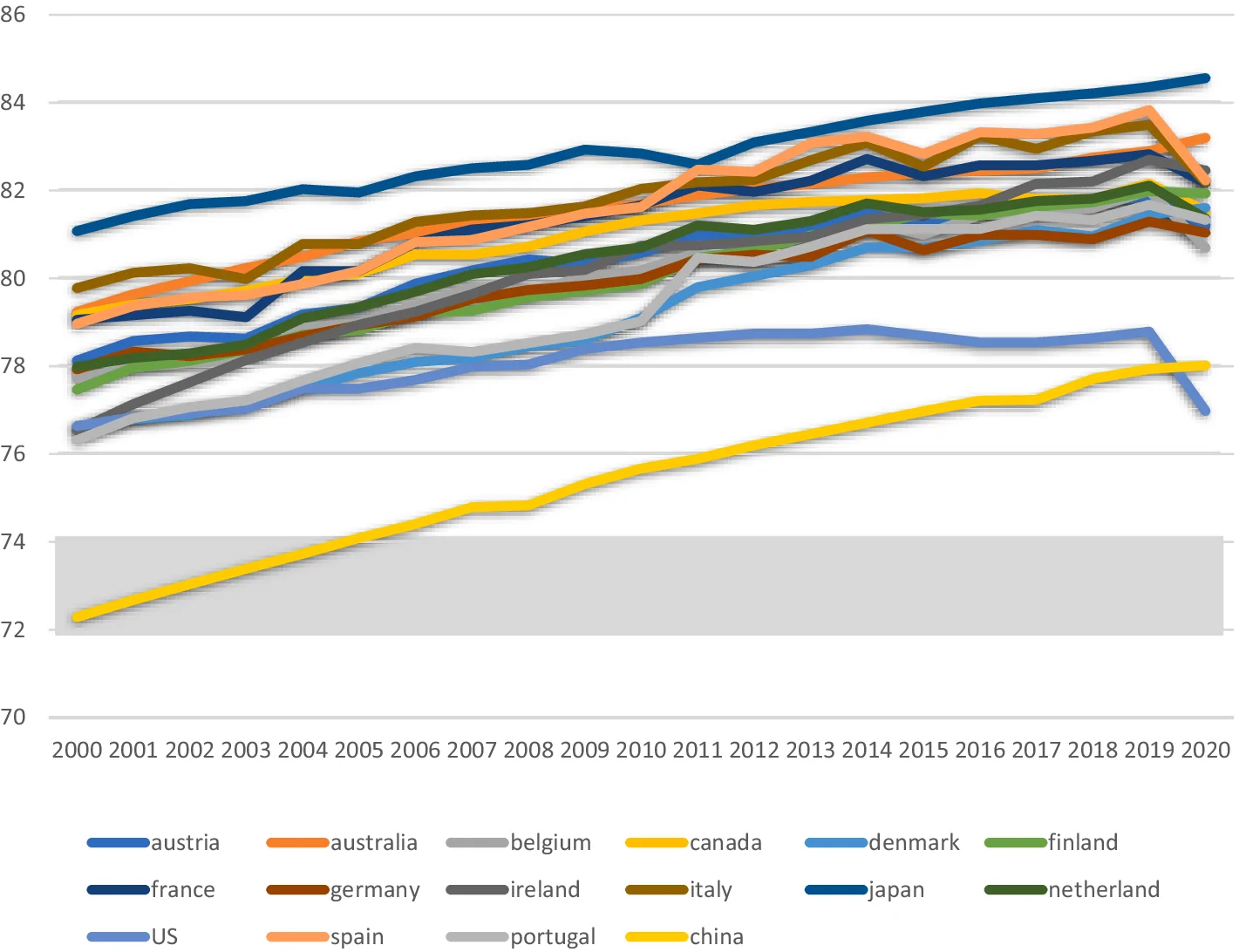
Variability in life expectancy across all selected countries from 2000–2020 (Source: World Development Indicators (World Bank); authors’ presentation).

## 2 Overview of previous research

In recent years, the relationship between the low-carbon economy transition, environmental policy stringency (EPSI), R&D expenditure, air quality, renewable energy consumption, and public health has drawn more and more attention, due to the increasing concerns about climate change and sustainable development (32, 33). In general, the existing research points to a conclusion that stricter environmental regulations promote cleaner production, technological development, and reduce environmental pollution (34). However, the evidence remains patchy as most studies tend to look at these relationships in isolation rather than in an integrated framework.

EPSI has been widely studied as a driver for improving environmental performance (35). OECD and emerging economies’ empirical evidence shows that good environmental policies foster investment in renewable energy and clean and green technologies that support the transition to low-carbon economic development (36, 37). But other studies suggest that the effectiveness of environmental directives depends on institutional standards, technological capabilities, and the degree of economic development, leading to heterogeneous policy outcomes across countries (38, 39). Within the low-carbon transition framework, EPSI can therefore be viewed as the policy dimension through which governments influence emissions control, cleaner production, and energy-system transformation.

Another major research area deals with R&D expenditure and broader technological capacity as important drivers of environmental sustainability (40). Previous studies consistently find that research investment, technological development, environmental patents, and clean technologies can contribute to reducing carbon emissions (CE), improving energy efficiency, and enhancing environmental quality (41). R&D expenditure can support the development and diffusion of more efficient technologies while helping firms adapt production processes and maintain productivity and competitiveness (42). However, the extent to which broader R&D capacity is associated with public-health outcomes remains less clearly established, particularly when environmental policy, energy transition, and air quality are considered simultaneously (43).

There has been a significant increase in research on the association between the shift to a low-carbon economy and public health in recent years (44). Research shows that reducing reliance on fossil fuels, boosting renewable energy, and enhancing energy efficiency not only combat climate change but also deliver substantial health co-benefits through decreased exposure to air pollution (13). These co-benefits include reduced mortality, increased labor productivity, and lower healthcare costs. However, most empirical studies consider environmental or health outcomes separately and provide little evidence on the interrelated relationships (8). In this context, renewable energy consumption represents an important energy-transition dimension of low-carbon development, while industrial-process carbon emissions reflect the environmental pressure associated with carbon-intensive industrial activity.

The existing literature provides useful insights, but there are several gaps in the research (45). First, extant studies mainly focus on the ultimate effect of EPSI on environmental performance, while the combined roles of environmental policy, renewable energy transition, technological capacity, industrial carbon pressure, and air quality in relation to public health remain relatively less studied (46). Second, there is limited literature that integrates environmental policy, R&D-based technological capacity, renewable energy, industrial carbon emissions, environmental quality, and public health into one analytical framework. Third, empirical works often use conventional estimation methods, which estimate average effects but ignore heterogeneous effects across countries with different environmental and economic conditions (47). Closing these gaps is crucial to comprehending the wider societal benefits of environmental policies and for developing effective strategies that simultaneously achieve low-carbon development, environmental sustainability, and public health (48).

## 3 Theoretical framework

The study rests on three complementary theoretical perspectives, explaining how the strictness of environmental policy and the transition toward cleaner energy systems can support the low-carbon economy transition and contribute to improved public health through technological progress, reduced environmental pressure, and better air quality. Together, these theories form the conceptual basis for understanding the relationships between environmental regulation, technological progress, environmental quality, and human well-being (30).

The Porter Hypothesis, formulated by Porter and van der Linde (49), suggests that environmental regulations, when properly designed, can stimulate creativity, improve resource efficiency, and enhance the long-term competitiveness of firms. The prevailing view is that environmental regulations lead to excessive compliance costs (50). However, the Porter Hypothesis argues that stringent environmental policies stimulate firms to invest in cleaner technologies, upgrade production processes, and develop environmentally friendly products. Innovation-driven improvements can offset the costs of regulation and reduce pollution and CEs (51, 52).

In this research, the EPSI is the main policy instrument to stimulate firms and industries to adopt green technologies and energy-efficient production systems. R&D expenses represent broader capabilities for national research, technology development, and innovation that can contribute to cleaner manufacturing processes, technology upgrades, and improved efficiency in resource use (53). Renewable energy consumption complements this policy dimension by capturing the energy-transition component of the low-carbon economy transition (54, 55). Therefore, the Porter Hypothesis provides a theoretical basis for examining how environmental policy stringency, research and technological capacity, and cleaner energy use are associated with environmental and public-health outcomes (31).

The Environmental Kuznets Curve (EKC) theory explains the dynamic relationship between the quality of the environment and economic development. The EKC demonstrates a curvilinear association between economic development and ecological deterioration. In the initial stages, economic growth increases, but then decreases as countries reach higher levels of income and adopt cleaner technologies, stronger environmental institutions, and more effective environmental regulations (56).

The theory suggests that technological progress, structural transformation, and stricter environmental policies will eventually decouple economic growth from environmental degradation (57). The EKC framework of the current study supports the hypothesis that stringent environmental policies, technological development, and greater reliance on renewable energy can reduce environmental pressure and improve air quality, thereby supporting the transition toward a sustainable low-carbon economy (58). Within this framework, industrial-process carbon emissions represent industrial carbon pressure rather than the transition itself, while EPSI and REC capture the policy and energy-transition dimensions, respectively.

According to Grossman’s (59) Health Production Theory, health is considered a form of human capital produced through investments in medical care, education, lifestyle, and environmental quality. The gains in general welfare and the reduction in people’s exposure to health risks are attributed to better environmental conditions, according to the theory. Therefore, the quality of the environment, especially clean air, is important for achieving better health outcomes.

In this study, stronger environmental policy stringency may contribute to improved air quality by encouraging cleaner production and limiting environmentally harmful activities, while greater renewable energy use may reduce dependence on more carbon-intensive energy sources. Lower environmental pressure and improved air quality can, in turn, reduce population exposure to pollution-related health risks and support higher life expectancy (60). R&D expenditure may further contribute through broader technological development, productivity improvements, and the diffusion of more efficient production technologies (61).

Thus, this study conceptualizes the low-carbon economy transition through two complementary dimensions: environmental policy stringency as the policy dimension and renewable energy consumption as the energy-transition dimension. R&D expenditure represents broader research and technological capacity, industrial-process carbon emissions capture industrial carbon pressure, and PM2.5 reflects air-quality conditions. Together, these factors provide a framework for examining how environmental policy, energy transition, technological capacity, environmental pressure, and healthcare conditions are associated with public health. This theoretical framework is integrated and allows for a comprehensive assessment of the environmental, technological, and health dimensions of sustainable development, while at the same time providing a solid conceptual framework for the empirical analysis (62).

Figure 3 presents the conceptual framework underpinning the study. It illustrates the low-carbon economy transition through its policy dimension, represented by EPSI, and its energy-transition dimension, represented by REC. R&D expenditure captures broader research and technological capacity, while industrial-process carbon emissions and PM2.5 represent environmental-pressure and air-quality conditions that are relevant to population health. These interconnected relationships ultimately provide the conceptual basis for examining life expectancy and sustainable development outcomes.

**Figure 3:**
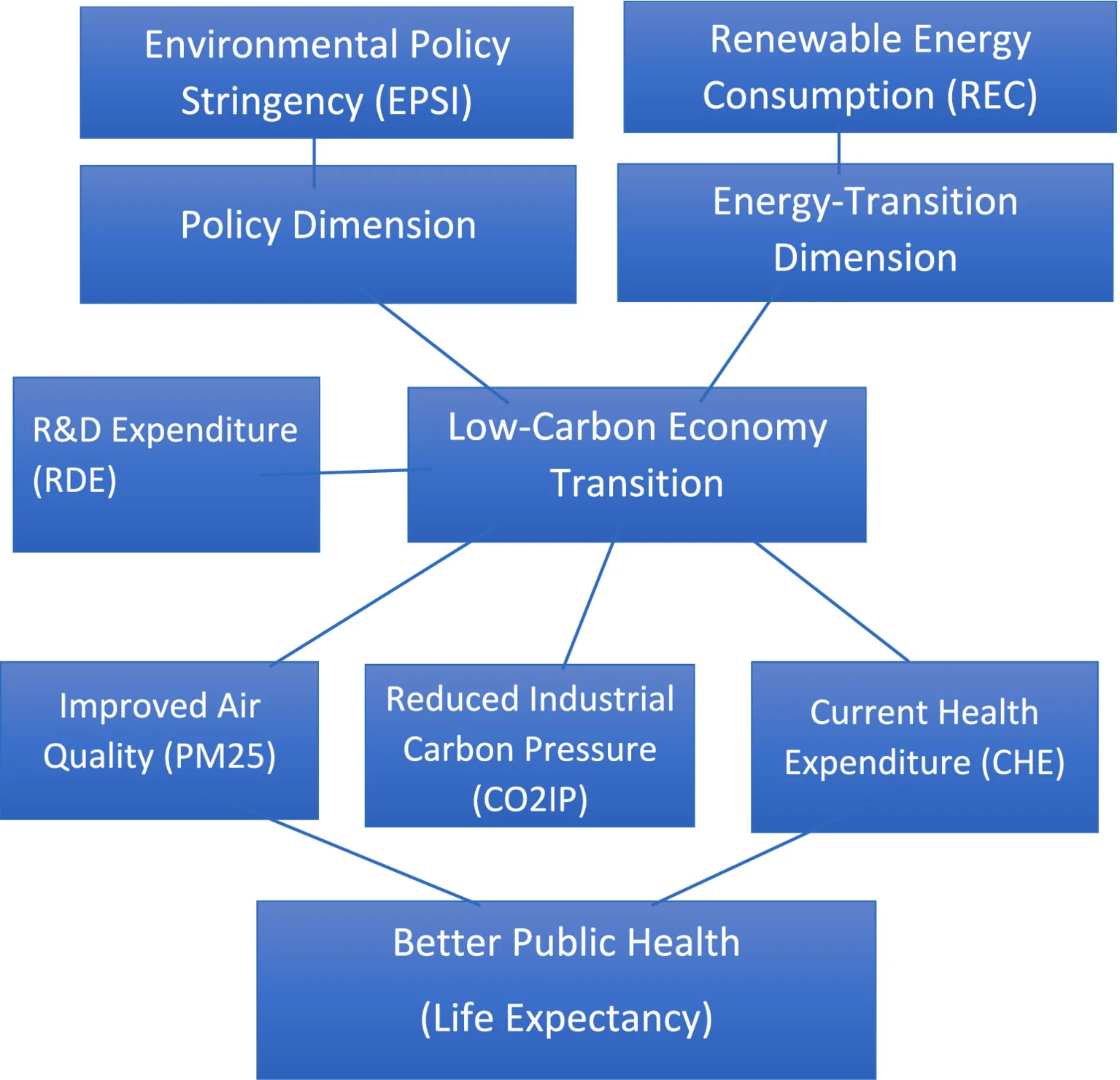
Conceptual diagram (Source: Authors’ conceptual framework based on the theoretical relationships discussed in the manuscript).

## 4 Data and method

The study uses panel data from 16 countries over the period of 2000 to 2020. The countries were selected according to heterogeneity in the implementation of environmental policy, technological innovation, energy transition, and performance of public health. These are all developed economies with a large share of global energy consumption and greenhouse gas emissions. Hence, they are particularly relevant for the study of the relationship between health outcomes, environmental regulation, and low-carbon development.

The analysis uses data from several internationally recognized databases to ensure consistency and reliability. The data on LE, REC, and CHE are obtained from the WDI published by the World Bank. Data on EPSI are collected from the Index obtained from the OECD databases. Research and development expenditure (RDE), measured as a percentage of GDP, is used as a broad proxy for technological and innovation capacity. RDE captures general research, technological development, and innovation capacity at the national level. It reflects the extent to which countries allocate economic resources to research and development activities that may contribute to technological progress, knowledge creation, productivity improvement, and the adoption of more efficient production processes. Accordingly, the empirical results for RDE are interpreted as reflecting the association between aggregate R&D investment and population health, with RDE representing broader national research and innovation capacity.

The low-carbon economy transition is conceptualized in this study as a multidimensional process rather than as a construct represented by a single empirical indicator. Two complementary dimensions are emphasized. The policy dimension is represented by the Environmental Policy Stringency Index (EPSI), which captures the strength of regulatory measures directed toward environmental protection, emissions control, and cleaner production. The energy-transition dimension is represented by renewable energy consumption (REC), measured as the share of renewable energy in total final energy consumption, which reflects the shift of the energy system toward cleaner and lower-carbon energy sources. Consequently, EPSI and REC capture complementary policy and energy dimensions of the broader low-carbon economy transition.

Air quality is measured by the annual mean PM 2.5 exposure from the World Bank. The industrial-process carbon emissions (CO2IP) are measured using carbon dioxide (CO₂) emissions from industrial processes, expressed in million tonnes of CO₂ equivalent (Mt CO₂e). The data are obtained from the World Development Indicators (WDI) of the World Bank. CO2IP captures the absolute scale of carbon emissions generated by industrial processes and is therefore treated as an indicator of industrial carbon pressure and the environmental burden associated with industrial activity. The same definition and data source are applied consistently across the selected countries and study period (2000–2020) to ensure comparability and replicability.

To investigate the connection between the low-carbon economy transition and public health, the baseline empirical model is specified in Equation 1.


$$\begin{array}{l} LE_{\mathrm{it}}=\alpha +\beta_{1}\mathrm{CO}2{\mathrm{IP}}_{\mathrm{it}}+\beta_{2}\mathrm{EPS}I_{\mathrm{it}}+\beta_{3}\mathrm{RD}E_{\mathrm{it}}+\beta_{4}\mathrm{PM}2.5_{\mathrm{it}}\\ +\beta_{5}\mathrm{RE}C_{\mathrm{it}}+\beta_{6}\mathrm{CH}E_{\mathrm{it}}+\mu_{i}+\lambda_{t}+\varepsilon_{\mathrm{it}} \end{array}$$ (1)

where:

-
LE represents public health measured by life expectancy.
-
CO2IP denotes the industrial-process carbon emissions.
-
EPSI denotes environmental policy stringency.
-
RDE represents research and development expenditure.
-
PM2.5 measures air quality.
-
REC denotes renewable energy consumption.
-
CHE represents current health expenditure.
-
$\mu_{i}$ captures country-specific fixed effects.
-
$\lambda_{t}$ captures time effects.
-
$\varepsilon_{\mathrm{it}}$ is the random error term.

The empirical analysis is based on a second-generation panel econometric framework given the potential for cross-sectional dependence induced by economic integration, globalization, and common environmental shocks.

The test is carried out in a series of stages. First, the CD test by Pesaran (63) is conducted to test the presence of cross-country dependence among the variables. Second, we apply the slope heterogeneity test of Pesaran and Yamagata (64) to check whether the slope coefficients are different across countries (65).

After confirming these panel characteristics, the stationarity properties of the variables are analysed with the CIPS unit root test that explicitly takes into account cross-sectional dependence. Then, we use the Westerlund (66) error-correction panel cointegration assessment to investigate the existence of a long-run equilibrium correlation among the parameters. This test can give a reliable inference in heterogeneous panels with cross-sectional dependence.

Given the panel structure of the dataset, the study employs the Fixed-Effects (FE) model with Driscoll–Kraay standard errors as the main estimation approach. The FE specification controls for unobserved, time-invariant country-specific characteristics that may be correlated with the explanatory variables, thereby reducing potential omitted-variable bias arising from cross-country heterogeneity. Driscoll–Kraay standard errors (DKSEs) are particularly appropriate in the current setting because they provide inference that is robust to heteroskedasticity, serial correlation, and general forms of cross-sectional dependence that may arise from common economic, environmental, technological, or policy shocks affecting the selected countries. This approach therefore provides more reliable statistical inference when conventional standard errors may be inappropriate (Table 1).

**Table 1:** Description of study data.

Variable	Symbol	Measurement	Source
Life expectancy (dependent variable)	LE	Years of life expectancy at Birth (total)	World Development Indicators (World Bank)
Industrial-process carbon emissions	CO2IP	Carbon dioxide emissions from industrial processes (Mt CO₂e); indicator of industrial carbon pressure and environmental burden	World Development Indicators (World Bank)
R&D expenditure	RDE	Research and development expenditure as a percentage of GDP; indicator of general research, technological development, and innovation capacity	World Development Indicators (World Bank) / OECD Database
Air quality	PM_2.5_	Population-weighted average exposure to PM₂.₅ (μg/m^3^)	World Development Indicators (World Bank) / WHO
Renewable energy consumption	REC	Renewable energy consumption as a percentage of total final energy usage; energy-transition dimension of the low-carbon economy transition	World Development Indicators (World Bank)
Current health expenditure	CHE	Current health expenditure as a percentage of GDP	World Development Indicators (World Bank)
Environmental policy stringency index	EPSI	Environmental Policy Stringency Index; policy dimension of the low-carbon economy transition	OECD Environmental Policy Stringency (EPS) Database

Authors’ compilation based on WDI (World Bank) and OECD Environmental Policy Stringency Database.

As a robustness check, the study additionally applies panel-corrected standard errors (PCSEs). The PCSE approach adjusts the estimated covariance matrix to account for heteroskedasticity and contemporaneous correlation across cross-sectional units, thereby providing an alternative inference framework for panel data characterized by interdependence among countries. Comparing the statistical significance, direction, and magnitude of the estimated coefficients under Driscoll–Kraay and PCSE inference allows the stability of the empirical relationships to be assessed under alternative treatments of the panel error structure. The combined use of FE with Driscoll–Kraay standard errors and PCSE therefore strengthens the robustness assessment of the reported findings.

## 5 Results and discussion

Table 2 displays descriptive statistics of study parameters for 336 observations from selected developed economies. The average LE is 80.179 years (from 72.288 to 84.560 years), which is a relatively high health outcome with moderate cross-country variation (SD = 2.143). The mean of industrial-process carbon emissions (CO2IP) is 92.361, and the standard deviation is 266.664, indicating substantial dispersion in the absolute scale of industrial carbon emissions across countries. The wide range (1.039–1553.099) further highlights marked cross-country heterogeneity in industrial carbon pressure and the environmental burden associated with industrial activity.

**Table 2:** Descriptive statistics of study variables.

Variable	Obs.	Mean	SD	Minimum	Maximum
LE	336	80.179	2.143	72.288	84.560
CO2IP	336	92.361	266.664	1.039	1553.099
RDE	336	2.116	0.732	0.698	3.734
PM2.5	336	14.402	9.457	4.744	52.727
REC	336	14.885	10.328	1.400	47.300
CHE	336	9.518	2.316	3.617	18.516
EPSI	335	2.697	0.852	0.222	4.889

Authors’ calculations are based on the study variables.

R&D expenditure (RDE) is on average 2.116% of GDP with relatively limited variation (SD = 0.732). This indicates that most selected economies allocate a relatively consistent share of their economic resources to research and development activities, reflecting broader national research, technological development, and innovation capacity. The average PM2.5 exposure is 14.402 μg/m3, but the maximum value of 52.727 μg/m3 indicates that even in developed economies, some countries still have relatively poor air quality. The average share of Renewable Energy Consumption (REC) in total final energy consumption is 14.885%. The high Standard Deviation (SD = 10.328) indicates a large gap in the adoption of renewable energy among countries. This variation also reflects important differences in the energy-transition dimension of the low-carbon economy transition across the sample. The average for current healthcare expenditure (CHE) is 9.518 percent of GDP, reflecting a high level of public investment in the healthcare systems. Finally, the mean value of the Environmental Policy Stringency Index (EPSI) is 2.697, with a minimum value of 0.222 and a maximum value of 4.889, indicating sizeable differences in the stringency of environmental regulations across the countries in the sample. These differences reflect heterogeneity in the policy dimension of the low-carbon economy transition. Overall, the descriptive statistics suggest that there is enough heterogeneity in all variables, providing an appropriate basis for panel econometric analysis.

The Pearson correlation coefficients of the study variables are presented in Table 3. The environmental policy stringency (EPSI) is significantly and positively correlated with life expectancy (r = 0.688), suggesting that countries with more stringent environmental policies tend to have better public health. Health care spending (r = 0.266) and RDE (r = 0.152) are positively correlated with life expectancy. The correlation between RDE and health is relatively weak at the bivariate level.

**Table 3:** Pearson correlation matrix.

Variable	LE	CO2IP	RDE	PM2.5	REC	CHE	EPSI
LE	1.000						
CO2IP	−0.479***	1.000					
RDE	0.152***	−0.070	1.000				
PM2.5	−0.582***	0.810***	−0.261***	1.000			
REC	0.032	−0.053	0.237***	−0.127**	1.000		
CHE	0.266***	−0.438***	0.422***	−0.566***	−0.021	1.000	
EPSI	0.688***	−0.226***	0.428***	−0.377***	0.229***	0.329***	1.000

Authors’ calculations. Note: *, **, and *** indicate statistical significance at the 10%, 5%, and 1% levels, respectively.

Several diagnostic tests were used to select the suitable econometric methodology before estimating the long-run relationships. Table 4 reports the Pesaran (63) cross-sectional dependence (CD) test, which indicates that all the variables are significantly dependent (*p* < 0.001). These results suggest that economic integration, globalization, diffusion of technology, and common environmental policies induce interdependence among the selected countries. Thus, first-generation panel estimators assuming independence across the cross-section would produce biased and inconsistent estimates.

**Table 4:** Cross-sectional dependence test.

Variable	CD Statistic	*p*-value
LE	47.780	<0.001
CO2IP	9.670	<0.001
RDE	18.260	<0.001
PM2.5	30.050	<0.001
REC	35.120	<0.001
CHE	38.950	<0.001
EPSI	41.360	<0.001

Authors’ calculations.

Table 5 also shows the slope heterogeneity test rejecting the null hypothesis of homogeneous slope coefficients. The results show that the Delta (6.163) and the Adjusted Delta (7.833) are both statistically significant at the 1 percent level, which confirms the fact that the effects of environmental policy, renewable energy, RDE, and other explanatory variables vary across countries. This is a natural result since developed economies are different in their institutional quality, environmental regulations, technological capabilities, and healthcare systems. Given the cross-sectional dependence and slope heterogeneity in the variables of the study, second-generation panel econometric techniques are more appropriate to estimate the relationships between the variables of the study.

**Table 5:** Slope heterogeneity test.

Test	Statistic	*p*-value	Decision
Delta	6.163	<0.001	Reject homogeneity
Adjusted Delta	7.833	<0.001	Reject homogeneity

Authors’ calculations.

The results of the Cross-sectionally Augmented IPS (CIPS) unit root test are shown in Table 6. Life expectancy is stationary at levels, implying an integration order of I(0). On the other hand, the variables CO2IP, RDE, PM_2.5_, REC, and CHE are stationary in first differences. These variables are therefore integrated of order one, I(1), while life expectancy and EPSI are integrated of order zero, I(0). CIPS results indicate that LE and EPSI are I(0), and CO2IP, RDE, PM_2.5_, REC, and CHE are I(1). In light of this combination of I(0) and I(1), Westerlund (66) Error Correction Model cointegration test for panel data is applied as another check for long-run co-movement. However, since the methodological approach is mainly geared toward non-stationary I(1) variables, its findings are taken with caution and viewed as evidence in favor of the existence of a long-run relationship rather than as conclusive evidence of long-run equilibrium for all parameters. The test is based on the error-correction specification. It allows for heterogeneous adjustment across countries and uses robust *p*-values to account for cross-sectional dependence. The robust *p*-values are particularly relevant in the present panel because the earlier cross-sectional dependence tests indicate significant interdependence among the countries. The estimated long-run relationship is specified in Equation 2.


$$\begin{array}{l} {\mathrm{LE}}_{\mathrm{it}}=\alpha_{i}+\beta_{1}\mathrm{CO}2{\mathrm{IP}}_{\mathrm{it}}+\beta_{2}{\mathrm{RDE}}_{\mathrm{it}}+\beta_{3}{\mathrm{PM}}_{2.5\mathrm{it}}\\ +\beta_{4}{\mathrm{REC}}_{\mathrm{it}}+\beta_{5}{\mathrm{CHE}}_{\mathrm{it}}+\beta_{6}{\text{EPSI}}_{\mathrm{it}}+\varepsilon_{\mathrm{it}} \end{array}$$ (2)

**Table 6:** CIPS panel unit root test.

Parameters	Level	1st Difference	Order of integration
LE	−3.027***	−5.064***	I(0)
CO2IP	−2.226	−3.938***	I(1)
RDE	−1.163	−3.894***	I(1)
PM2.5	−2.305	−4.802***	I(1)
REC	−2.470	−4.851***	I(1)
CHE	−1.829	−3.351***	I(1)
EPSI	−2.981***	−4.923***	I(0)

***p < 1%. Authors’ calculations.

Where i denotes country and t denotes year; αᵢ represents country-specific effects, β₁–β₆ are the long-run coefficients, and εᵢₜ is the error term.

Considering the varying orders of integration observed from the CIPS test, the findings of Westerlund are taken as evidence supporting long-run comovement and not as conclusive evidence of equilibrium between the variables. The findings of the Westerlund cointegration test, shown in Table 7, offer strong evidence of long-run comovement among the variables. Both Group-tau (Gt) and Panel-tau (Pt) statistics are statistically significant at the 1 percent level, and the robust *p*-values for all four statistics are still highly significant. These results reject the null hypothesis of no cointegration under the reported test statistics and indicate that life expectancy, environmental policy stringency, renewable energy consumption, industrial-process carbon emissions, air quality, RDE, and healthcare expenditure exhibit long-run comovement despite short-run fluctuations. Thus, the findings provide a reasonable basis for proceeding with robust panel estimation, while the long-run interpretation is maintained cautiously in light of the mixed I(0)/I(1) structure.

**Table 7:** Westerlund panel cointegration test.

test Statistic	Statistic	Z-value	*p*-value	Robust *p*-value
Group-tau (Gt)	−4.566	−7.201	0.000***	0.000***
Group-alpha (Ga)	−8.944	3.653	1.000	0.000***
Panel-tau (Pt)	−18.973	−8.051	0.000***	0.000***
Panel-alpha (Pa)	−10.209	1.382	0.916	0.000***

***p < 1%. Authors’ calculations.

The positive and statistically significant coefficient of Environmental Policy Stringency (EPSI) (β = 0.6760, *p* < 0.01) as shown in Table 8 indicates that stronger environmental regulations are associated with higher life expectancy in the selected developed economies. This finding is consistent with previous empirical evidence showing that stringent environmental policies can improve environmental performance by encouraging cleaner production, technological upgrading, and investment in environmentally friendly technologies (34, 35). The result also supports the broader evidence that effective environmental policies can promote renewable energy and green technologies (36). The positive association observed in this study may therefore reflect the health co-benefits of stronger environmental governance, including reductions in environmental risks and improvements in environmental quality (67). Within the present study framework, EPSI represents the policy dimension of the low-carbon economy transition, and its positive coefficient suggests that stronger environmental-policy frameworks are positively associated with population health.

**Table 8:** Fixed-effects model with Driscoll–Kraay standard errors.

Variable	Coefficient	Driscoll–Kraay Std. error	t-statistic	*p*-value	95% Confidence interval
CO2IP	0.0046***	0.0003	13.37	0.000	[0.0039, 0.0053]
RDE	0.0822	0.2046	0.40	0.692	[−0.3446, 0.5090]
PM2.5	−0.0233	0.0330	−0.71	0.489	[−0.0921, 0.0456]
REC	0.1517***	0.0194	7.82	0.000	[0.1112, 0.1922]
CHE	0.1733*	0.0946	1.83	0.082	[−0.0241, 0.3708]
EPSI	0.6760***	0.1671	4.04	0.001	[0.3274, 1.0247]
Constant	74.1868***	0.9545	77.72	0.000	[72.1957, 76.1779]
Observations	336				
Number of countries	16				
Within R-sq.	0.8129				
F-stat.	525.47				
Prob > F	0.0000				

***p < 1% and *p < 10% levels. Authors’ calculations.

Industrial-process carbon emissions (CO2IP) exhibit a positive and statistically significant coefficient in the baseline specification (β = 0.0046, *p* < 0.01). Since CO2IP measures the absolute scale of emissions from industrial processes, this positive association should be interpreted cautiously and does not imply that higher industrial emissions improve population health. The unexpected sign may partly reflect differences in the scale and structure of industrial activity across the selected economies, where countries with larger industrial sectors may simultaneously possess higher incomes, stronger healthcare systems, more advanced infrastructure, and greater institutional capacity. In addition, time-varying socioeconomic and structural factors not fully captured by the model may contribute to the estimated association. Accordingly, the CO2IP coefficient is interpreted as a conditional association between industrial carbon pressure and life expectancy rather than as evidence of a beneficial health effect of industrial emissions. Its stability is further assessed through the robustness specification.

REC also has a positive and statistically significant relationship with life expectancy (β = 0.1517, *p* < 0.01). This finding is consistent with previous research proposing that the transition from fossil fuels toward renewable energy can generate important health co-benefits through reduced pollution exposure and improved environmental quality (13). The result is also consistent with the literature identifying renewable energy as an important component of low-carbon development (68). Within the conceptual framework of this study, REC represents the energy-transition dimension of the low-carbon economy transition. Its positive association with life expectancy therefore suggests that a greater renewable share in final energy consumption is associated with improved population-health outcomes. However, the magnitude of the association should be interpreted as a conditional relationship within the multivariate panel model rather than as evidence of a direct causal health effect.

In contrast, R&D expenditure (RDE) is positive but statistically insignificant (β = 0.0822, *p* = 0.692). This finding suggests that aggregate R&D expenditure does not have a statistically identifiable contemporaneous association with life expectancy after controlling for environmental, energy, and healthcare factors. One possible explanation is that RDE captures broad national research, technological development, and innovation capacity encompassing diverse sectors and activities, whose benefits may differ in their relevance to population health. Moreover, the effects of R&D investment may emerge with considerable time lags through technological development, diffusion, productivity improvements, and adoption. Differences across countries in the composition, efficiency, and utilization of R&D expenditure may also weaken its contemporaneous association with life expectancy. Accordingly, the insignificant coefficient does not imply that research and technological development are unimportant, but rather that aggregate R&D expenditure does not show a statistically robust direct association with population health in the baseline specification.

PM₂.₅ has the expected negative coefficient (β = − 0.0233), but its effect is statistically insignificant (*p* = 0.489). The negative sign is broadly consistent with the established literature linking particulate-matter exposure with adverse health outcomes. However, the lack of statistical significance differs from studies reporting stronger health effects of air pollution. This divergence may reflect several characteristics of the present sample and specification. Life expectancy is a broad and slowly changing indicator of population health, whereas the adverse consequences of PM₂.₅ exposure may be more directly reflected in respiratory morbidity, cardiovascular mortality, premature mortality, or other disease-specific outcomes. In addition, relatively limited within-country variation in pollution exposure among some of the selected economies, together with the simultaneous inclusion of EPSI and REC, may reduce the precision with which the independent association of PM₂.₅ is estimated. Therefore, the statistically insignificant coefficient should not be interpreted as evidence that particulate pollution has no health consequences, but rather that an independent association is not identified in the present specification.

Healthcare expenditure (CHE) is positively associated with life expectancy and is weakly significant at the 10% level (β = 0.1733, *p* = 0.082). This finding is broadly consistent with the health-production perspective, which recognizes healthcare investment as an important input into population health. Nevertheless, its weaker statistical significance compared with EPSI and REC suggests that differences in life expectancy across the selected countries cannot be attributed to healthcare expenditure alone and may also depend on environmental quality, energy structure, and environmental governance.

Table 9 presents the robustness analysis using the Prais–Winsten regression with panel-corrected standard errors (PCSEs). The results provide partial support for the baseline findings. EPSI remains positive and statistically significant (β = 0.6907, *p* < 0.01), while REC also retains a positive and significant association with life expectancy (β = 0.0111, *p* < 0.05). CHE remains positive and statistically significant at the 5% level (β = 0.1492, *p* < 0.05). PM₂.₅ becomes negative and statistically significant (β = −0.1087, *p* < 0.01), providing stronger evidence of an adverse association between particulate-matter exposure and life expectancy. In contrast, CO2IP (β = −0.0004, *p* = 0.582) and RDE (β = 0.0330, *p* = 0.834) remain statistically insignificant. Overall, the PCSE results reinforce the positive associations of EPSI and REC with life expectancy, while also showing a robust negative association for PM₂.₅. The evidence for CO2IP and RDE remains statistically inconclusive.

**Table 9:** Robustness analysis: Prais–Winsten regression, panel-corrected standard errors (PCSEs).

Variable	Coefficient	Panel-corrected SE	z-statistic	*p*-value	95% Confidence interval
CO2IP	−0.0004	0.0008	−0.55	0.582	[−0.0019, 0.0011]
RDE	0.0330	0.1576	0.21	0.834	[−0.2760, 0.3419]
PM₂.₅	−0.1087***	0.0225	−4.84	0.000	[−0.1527, −0.0647]
REC	0.0111**	0.0048	2.31	0.024	[0.0017, 0.0205]
CHE	0.1492**	0.0749	1.99	0.046	[0.0026, 0.2958]
EPSI	0.6907***	0.1165	5.93	0.000	[0.4624, 0.9190]
Constant	81.2545***	1.0481	77.53	0.000	[79.2003, 83.3087]
Observations	336				
Number of countries	16				
R-sq.	0.9952				
Wald χ^2^ (6)	126.21				
Prob > χ^2^	0.0000				
ρ	0.8789				

*** and ** indicate statistical significance at the 1 and 5% levels, respectively. Authors’ calculations.

## 6 Conclusion and policy implications

### 6.1 Conclusion

This study investigates the long-run relationships between the low-carbon economy transition, represented through environmental policy stringency and renewable energy consumption, R&D expenditure, air quality, industrial-process carbon emissions, healthcare expenditure, and life expectancy for 16 developed economies over the period 2000–2020. Results reveal that environmental policy stringency and renewable energy usage are positively and significantly related to life expectancy, while healthcare expenditure has a weak positive relation with life expectancy. Industrial-process carbon emissions are positively significant in the baseline model, whereas R&D expenditure and PM₂.₅ are statistically insignificant. The baseline results are partially supported by the robustness analysis using Prais–Winsten regression with panel-corrected standard errors (PCSEs). The associations of EPSI and REC with life expectancy remain positive and statistically significant, while CHE also remains positively significant. PM₂.₅ becomes negative and statistically significant in the robustness model, whereas CO2IP and RDE are statistically insignificant. The change in significance of CO2IP across specifications indicates that its baseline association is not robust, while the negative PM₂.₅ result provides stronger evidence of an adverse relationship between particulate-matter exposure and life expectancy. Overall, the results indicate that environmental policy stringency and renewable energy depletion are the most consistently supported dimensions of the low-carbon economy transition in relation to population health, while the evidence for industrial carbon pressure and aggregate R&D expenditure remains less robust. However, these findings are statistical associations and should be interpreted with caution as they do not imply causality.

### 6.2 Policy implications

The empirical results provide a number of policy considerations for governments, environmental agencies and international organizations. The implications should be seen as policy recommendations derived from observed statistical associations, and not as prescriptions based on established causal effects.

First, policymakers could consider maintaining consistency and transparency in environmental policy frameworks. The positive and statistically significant connection between EPSI and life expectancy suggests that environmental policy stringency is important for the broader relationship between environmental governance and population health. As the policy dimension of the low-carbon economy transition, EPSI is also one of the most consistently supported variables across the baseline and robustness specifications. Such measures as carbon pricing, emissions standards, pollution taxes, and emissions trading mechanisms may thus be seen as parts of broader frameworks of environmental policy. But the design has to take into account country-specific institutional and economic conditions.

Secondly, there might be more focus on the development of renewable energy. The positive and statistically significant correlation between REC and life expectancy, which remains significant in the PCSE robustness analysis, points toward a relatively stable association between renewable energy consumption and population health in the sample. As the energy-transition dimension of the low-carbon economy transition, REC provides consistent empirical support for the relevance of cleaner energy structures to population health. Therefore, policymakers might want to consider policies that facilitate the adoption of renewable energy, including investment in renewable energy infrastructure, improvements in grid capacity, and incentives for cleaner energy technologies.

Third, coordination of environmental and public-health policy might be improved. The coefficient on PM2.5 is negative in both specifications; although it is statistically insignificant in the baseline model, it becomes negative and statistically significant in the PCSE robustness analysis. This pattern provides stronger evidence that higher particulate-matter exposure is associated with lower life expectancy when the panel error structure is treated differently. Therefore, pollution control, emissions management and air-quality monitoring remain relevant components of coordinated environmental and public-health policy. This is especially true given the environmental differences among countries.

Fourth, health-care spending should remain a part of broader population-health strategies. CHE is positively related to life expectancy and is weakly significant in the baseline model and statistically significant at the 5 percent level in the PCSE specification. This finding suggests that healthcare expenditure is relevant to cross-country differences in life expectancy in the sample, although the magnitude and significance of this relationship suggest that healthcare spending alone does not explain population-health differences. Hence, policymakers may consider healthcare investment along with environmental, energy, and socioeconomic policies.

Fifth, the results of R&D expenditure should be interpreted with caution for policy. RDE is not statistically significant in either the baseline or robustness estimations. The measure captures aggregate national R&D expenditure and therefore reflects broad research, technological development, and innovation capacity. The results do not provide sufficiently strong evidence to conclude that higher aggregate R&D expenditure is systematically associated with higher life expectancy. Therefore, policy conclusions concerning R&D should remain limited to the evidence provided by the aggregate measure, while future strategies may place greater emphasis on the composition, efficiency, and health-relevant application of R&D investment.

Industrial-process carbon emissions should also be interpreted cautiously in policy terms. CO2IP is positive and statistically significant in the baseline model but becomes negative and statistically insignificant in the PCSE robustness specification. This lack of stability indicates that the relationship between absolute industrial carbon pressure and life expectancy is not robust across specifications. Accordingly, the present results do not provide sufficiently consistent evidence for drawing direct policy prescriptions from the CO2IP coefficient alone.

Finally, the evidence of slope heterogeneity suggests that policy responses should not necessarily be one size fits all. The relationship between environmental policy, energy use, environmental conditions and life expectancy may differ across countries, depending on their institutional capacity, technological capabilities, energy structure, health care systems and environmental conditions. This may lead policymakers to consider country-specific policy designs that take into account local economic and institutional circumstances.

Collectively, the policy implications suggest greater coordination among environmental regulation, renewable energy development, health care investment and environmental monitoring. The strongest and most consistent policy-relevant evidence concerns environmental policy stringency and renewable energy consumption, while the findings for PM2.5 and healthcare expenditure provide additional, but comparatively specification-sensitive, support. Direct policy conclusions from CO2IP and RDE should remain cautious because their estimated relationships are not consistently supported across specifications. However, these recommendations should be read in light of the empirical limitations of the study and the non-causality of the estimated relations.

## 7 Limitations and future directions

The model presented in this study does not include some socio-economic, demographic and institutional determinants of life expectancy, and therefore potential endogeneity and omitted-variable bias cannot be totally ruled out. The results should therefore be understood as associations of a statistical nature and not as causal effects. Future studies may also involve other factors such as governance quality, environmental regulation, climate finance, digital transformation, financial development, urbanisation and education. It can also investigate nonlinear and threshold effects with the aid of panel threshold models, dynamic panel techniques, PVAR or PSEM for further insights into the relationships among environmental policy, innovation, environmental quality and public health. The primary measure of public health in this study is life expectancy. It provides a general indicator of population health and longevity but may not capture specific health outcomes associated with environmental conditions, such as respiratory diseases, morbidity, or premature mortality.

## Data Availability

The original contributions presented in the study are included in the article/supplementary material, further inquiries can be directed to the corresponding author.
